# Teprotumumab, an insulin-like growth factor-1 receptor antagonist antibody, in the treatment of active thyroid eye disease: a focus on proptosis

**DOI:** 10.1038/s41433-018-0321-y

**Published:** 2018-12-21

**Authors:** Raymond S. Douglas

**Affiliations:** Cedars Sinai Medical Center Los Angeles and Zhongshen Ophthalmic Center, Guangzhou, China

## Abstract

Thyroid eye disease is a disabling autoimmune disease associated with orbital inflammation and tissue remodeling which can result in significant proptosis, leading to visual alterations and is potentially sight threatening. Current evidence indicates that autoantibodies to the insulin-like growth factor 1 receptor (IGF-1R), along with the thyroid-stimulating hormone receptor (TSHR), mediate the pathogenesis in susceptible individuals. Teprotumumab, a monoclonal IGF-1R antagonist, has demonstrated previously in a 24 week, randomized, controlled trial to produce significant changes in composite outcomes of proptosis and clinical activity score as compared with placebo. Further examination of the proptosis results reported here, indicate that the proptosis outcome (≥ 2 mm reduction) was met in 71.4% of the teprotumumab-treated patients as compared with 20% of the placebo-treated patients (*p* < 0.001). Additionally, the proptosis benefit was observed early in the trial (study week 6), and all individual patients demonstrated some benefit at week 24. Improvement was noted among smokers, non-smokers, men and women, and particularly those with higher levels of proptosis at baseline. The level of proptosis reduction with teprotumumab reported here is similar to that seen with decompression surgery. If these results are confirmed in the ongoing Phase 3 trial, teprotumumab will offer an alternative to surgery and its associated complications.

## Introduction

**Fig. 1:**
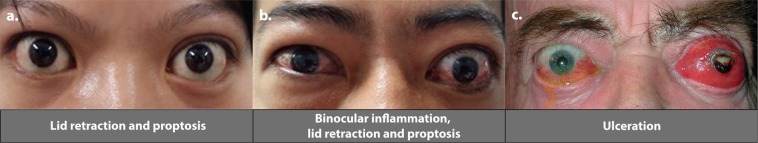
Diverse presentations of thyroid eye disease [24, 25]

Thyroid eye disease (TED), also commonly known as Graves’ Ophthalmopathy, Graves’ Orbitopathy and Thyroid-Associated Ophthalmopathy is a debilitating autoimmune disease often associated with orbital inflammation, fibrosis, and fat expansion. In more severe cases there is permanent facial disfigurement and optic nerve involvement, which can be sight threatening. Currently no approved pharmacotherapies exist. The mainstay of treatment is observation and steroids for more severe cases, but currently there is no disease modifying therapy. Based on Rundle’s pioneering observations in 1945, there is a potential window of opportunity to treat TED patients during the initial inflammatory presentation of the disease, termed active disease (Fig. 1b). The time period for active disease varies but typically occurs over 1–3 years [1–3]. Once the disease progresses to inactive or fibrotic disease (Fig. 1a), surgical options are considered.

TED is a heterogeneous disease with diverse phenotypes, but proptosis is one of the most prevalent aspects of the disease (Fig. 1). Proptosis reflects the orbital tissue infiltration and expansion posterior to the eye which then causes anterior displacement. Excessive proptosis can lead to ocular sequelae, such as corneal exposure, possible ulceration, and other visual alterations (Fig. 1c). There is also a profound change in facial appearance due to tissue infiltration. The manifestations taken together can cause marked psychosocial distress for TED patients, leading to a marked reduction in overall quality of life [4, 5]. Proptosis can occur in a range of severity from mild (defined as < 3 mm) to moderate–severe proptosis (defined as ≥ 3 mm) as compared with the upper limit of normal for each race/sex or the patient’s baseline, if available [3].

**Fig. 2:**
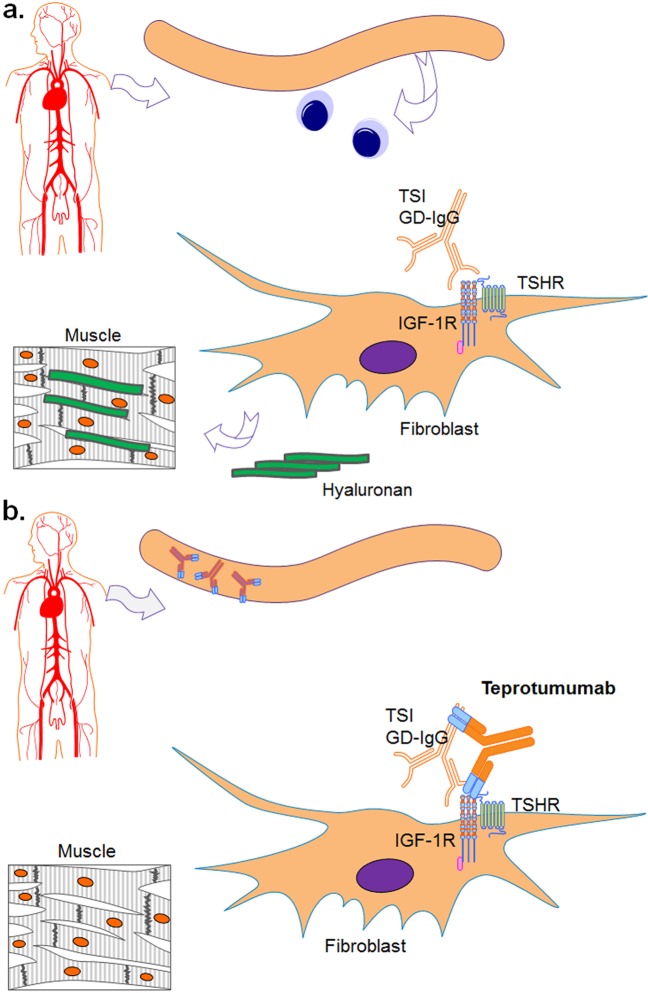
**a** Pathogenic autoantibodies stimulating the orbital fibroblasts resulting in production of hyaluronan and giving rise to symptoms of thyroid eye disease. **b** Teprotumumab (an IGF-1R antagonist) blocks the stimulatory effects of pathogenic autoantibodies on the orbital fibroblasts TSI thyroid stimulating immunoglobulins, GD-IgG Graves’ disease immunoglobulins, TSHR thyroid stimulating hormone receptor, IGF-1R insulin-like growth factor-1 receptor

The pathogenesis of TED remains poorly understood; the current theory is that autoantibodies to the thyroid-stimulating hormone receptor (TSHR) play a pivotal role in the pathogenesis of TED. Autoantibodies to TSHR alone, however, do not explain the disease presence in TED patients who are euthyroid or hypothyroid, suggesting that there might be another receptor at play in the pathogenesis of TED, such as the insulin-like growth factor 1 receptor (IGF-1R) [6]. Mounting evidence for this includes the overexpression of IGF-1R in TED, the presence of anti-IGF-1R antibodies in TED patients that are able to block signaling in the orbital fibroblasts, and the production of hyaluronan by the orbital fibroblasts in TED patients induced by both thyroid stimulating immunoglobulins (TSIs) and IGF-1 [7–10]. Further, Tsui et al. found evidence of the existence of a physical, functional complex comprising TSHR, and IGF-1R in the orbital fibroblasts of TED patients [11]. These data suggest that TED is due to the upregulation of the TSHR/IGF-1R complex in orbital fibroblasts of susceptible individuals leading to accumulation of glycosaminoglycans, including hyaluronan within the orbit and an expanded volume of fat and muscle adjacent to the eye (Fig. 2a) [7, 8, 11, 12].

There is currently no approved pharmacotherapy available for the treatment of TED, which presents an important unmet medical need. Many patients present with a mild course of disease and can be treated symptomatically and monitored for worsening. Patients with moderate-to-severe active disease may receive immunosuppressive therapy aimed at reducing the inflammation. However, oral and intravenous steroids do not reverse the underlying pathophysiology and they may temporarily mask the symptoms of the disease without considerable effect on the disease progression including proptosis. Furthermore, lengthy use of steroid therapy has considerable side effects. Steroid sparing agents and human monoclonal antibodies approved for other inflammatory indications have been used in TED, but these agents have not demonstrated disease-modifying efficacy [13–15]. In the absence of effective pharmacotherapies, the only option left for patients is eventual surgery once the inflammatory process subsides.

Teprotumumab, a fully human monoclonal IGF-1R antagonist, is currently in development for the treatment of active TED. Teprotumumab interrupts the pathologic actions of the IGF-1R and possibly TSHR/IGF-1R signaling complex (the molecular target) (Fig. 2b). A recently completed Phase 2 clinical study demonstrated that teprotumumab modified the disease endpoint in active TED patients. Results from the 24-week randomized, placebo-controlled study in TED patients with active, moderate–severe disease indicate a statistically significant reduction in the primary composite endpoint (defined as proptosis [≥ 2 mm reduction] and clinical activity score [CAS] ≥ 2 point reduction on 7 point scale) versus placebo [16]. The objective of the present analysis is to analyze the proptosis findings of the teprotumumab Phase 2 study.

## Materials and methods

**Fig. 3:**
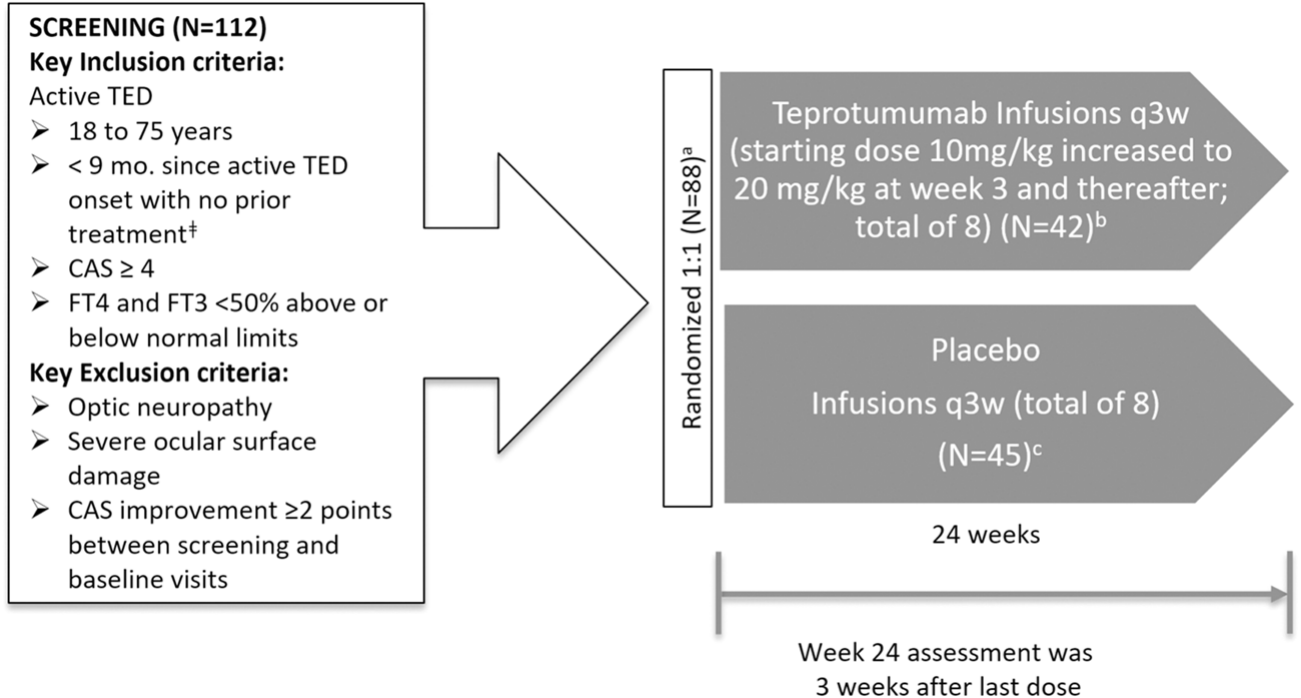
Study design. ^ǂ^Excluding local supportive measures and oral steroids if the maximum cumulative dose is less than 1000 mg methylprednisolone or equivalent. There must be at least 6 weeks between last administration of steroids and study randomization. ^a^One patient underwent randomization and withdrew prior to dosing. ^b^In total, 37 patients completed therapy, 5 discontinued teprotumumab due to an adverse event. ^c^In total, 39 patients completed therapy, 6 discontinued placebos (one had an adverse event and five had other reason)

The methodology of the trial has been previously described [16] and is summarized here (Fig. 3). The trial was a multicenter, double-masked, placebo-controlled Phase 2 study of teprotumumab administered every 3 weeks in patients with active moderate–severe TED. Patients were randomly assigned to either teprotumumab or placebo, stratified by smoking status.

For assessment of proptosis, the same Hertel exophthalmometer instrument and intercanthal distance was used at each time point. Every effort was made for the same observer to conduct the assessment on each occasion. The Hertel values were measured for each eye at all time points. For proptosis analysis, a repeated-measures mixed model was fit to the individual change from baseline scores, incorporating the baseline score, smoking status, treatment group, time, time by treatment, and time by baseline score interaction. This post-hoc analysis was conducted to better elucidate the proptosis outcome (reduction ≥ 2 mm) at 24 weeks in sub-groups receiving teprotumumab as compared with placebo.

## Results

As previously reported by Smith et al., 88 subjects were randomized, of whom 87 (42 in the teprotumumab group and 45 in the placebo group) received at least one treatment dose and were included in the intent-to-treat (ITT) analysis [16]. Seventy-six subjects (37 in the teprotumumab group and 39 in the placebo group) completed 24 weeks of treatment. Most demographics and baseline characteristics, including proptosis, clinical activity score (CAS), and Graves’ Ophthalmopathy Quality-of-Life (GO-QOL) combined subscales, were similar between the two groups, with the exception of diplopia, which occurred more frequently in the teprotumumab group. Despite stratification by smoking status, there were more smokers in the placebo group compared with the teprotumumab group (18 vs. 11).

### Proptosis outcome (≥2 mm reduction) results

**Fig. 4:**
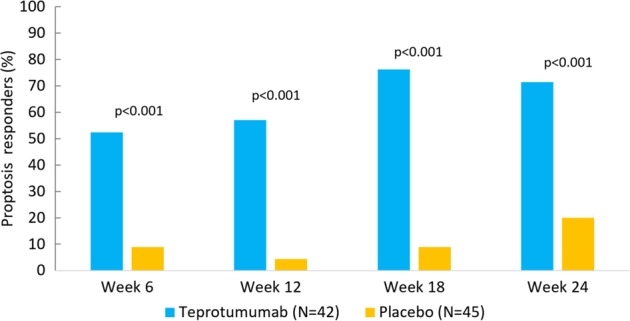
Proptosis responders (percent of patients with a decrease ≥  2 mm)

**Fig. 5:**
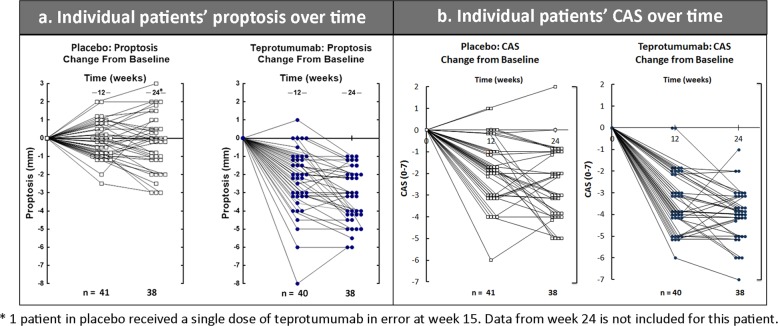
Individual patients’ proptosis and CAS over time

In total, 71.4% (30/42) teprotumumab-treated and 20% (9/45) placebo-treated patients achieved the proptosis outcome at week 24 (ITT analysis; *p* < 0.001) (Fig. 4). There was an early response to teprotumumab with ~50% of teprotumumab-treated patients reaching the proptosis outcome at week 6 (two infusions). Utilizing a repeated-measures mixed model, the least square (LS) mean reduction from baseline was significantly different from placebo at weeks 6, 12, 18, and 24 (*p* < 0.001) [16]. The mean reduction from baseline was −2.95 mm (standard error [SE] 0.266) at 24 weeks. Individual proptosis and CAS plots demonstrate that patients treated with teprotumumab had improved endpoints compared with placebo at all evaluation times (Fig. 5). Each patient with available data treated with teprotumumab had some degree of reduction of proptosis (Fig. 5a) and CAS (Fig. 5b) at week 24.

To further explore the response at week 24, subjects were classified into one of four response categories at week 24:

- High response: a reduction in proptosis of ≥ 3 mm from baseline
- Response: a reduction in proptosis of 2 to < 3 mm from baseline
- Low response: a reduction in proptosis of 1 to < 2 mm from baseline
- No response: a reduction in proptosis < 1 mm

At 24 weeks, a high response (reduction of ≥ 3 mm) was achieved in 54.8% of teprotumumab-treated patients compared with 8.9% of placebo-treated patients; 0% of teprotumumab-treated patients had no response compared with 48.9% of placebo-treated patients (Table 1).

**Table 1:** Proptosis graded response at week 24

	Placebo (*N* = 45)		Teprotumumab (*N* = 42)	
	No. of subjects	%	No. of subjects	%
High response	4	8.90%	23	54.80%
Response	5	11.10%	7	16.70%
Low response	8	17.80%	8	19.00%
No response	22	48.90%	0	0.00%
Missing	6	13.30%	4	9.50%
Total	45	100.00%	42	100.00%

**Fig. 6:**
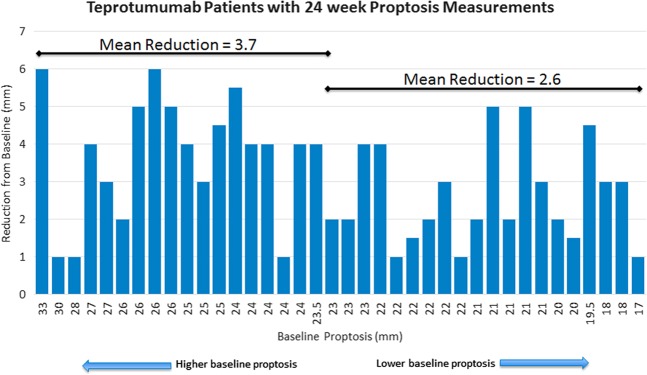
Proptosis reductions according to baseline proptosis measurements. Note: 1 patient did not receive the full course of treatment but had measurements at week 24

**Fig. 7:**
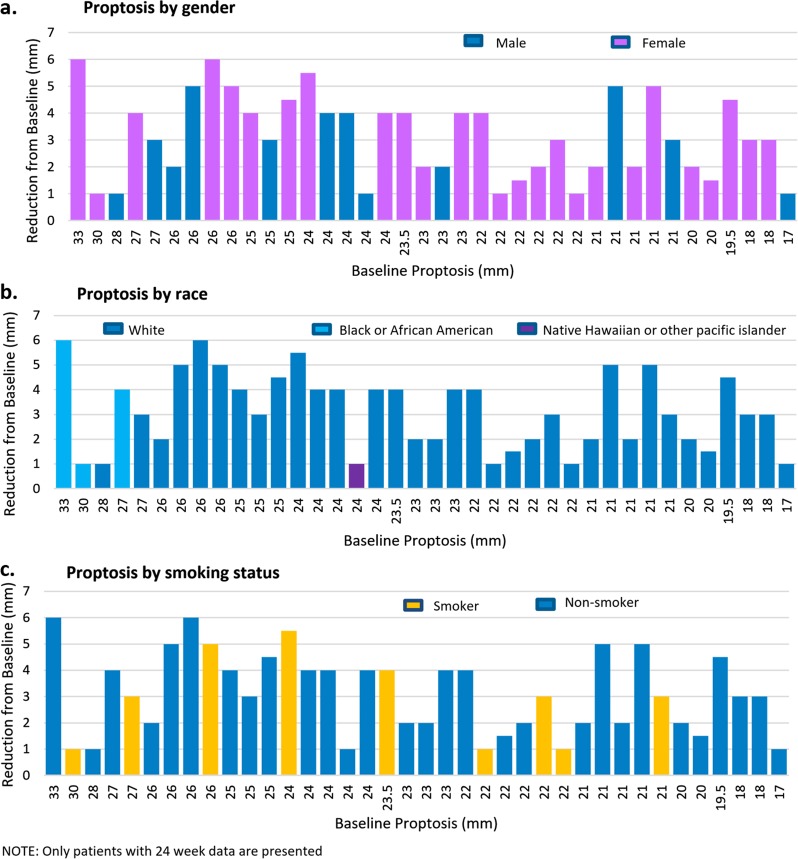
Proptosis response with teprotumumab stratified by race, gender and smoking status. Note: 1 patient did not receive the full course of treatment but had measurements at week 24

**Fig. 8:**
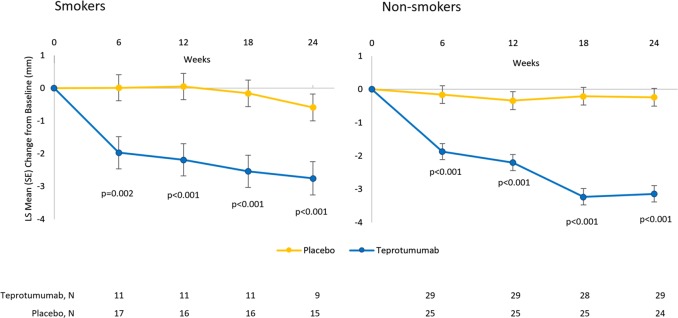
Reduction in Proptosis in smokers and non-smokers

An analysis of proptosis response according to the baseline proptosis measurement demonstrated that responses were greater with a higher baseline proptosis measurement; patients with higher baseline proptosis seem to have greater improvement with teprotumumab though both groups had meaningful reductions (Fig. 6). Reduction in proptosis with teprotumumab treatment is shown by gender, race (overwhelming majority of study population was white), and smoking status in Fig. 7. The majority of study participants were female; both males and females showed meaningful improvement in proptosis (−2.83 mm [males], −3.29 [females]). Both smokers and non-smokers had significant proptosis reductions from baseline at each timepoint (Figs. 7 and 8).

### Safety

As previously reported, adverse events that occurred in > 5% of patients on teprotumumab (and greater than placebo) were nausea (19%), muscle spasms (19%), diarrhea (14%), hyperglycemia (12%) and alopecia, dry skin, dysgeusia, headache, paresthesia, hearing impairment, and weight loss (7% each) [16]. Five subjects in the teprotumumab group experienced serious adverse events (SAEs) of diarrhea, inflammatory bowel disease, Escherichia sepsis, suspected Hashimoto’s encephalopathy, and urinary retention, of which suspected Hashimoto’s encephalopathy and diarrhea were considered possibly related to study drug. No deaths occurred during the study.

## Discussion

When active TED progresses, surgery is often required for proptosis and its associated findings of diplopia, lid retraction, or to debulk the eyelid. Proptosis reduction has been resistant to therapy, and agents in use have not demonstrated significant effect on proptosis [13–15, 17, 18]. In the published phase 2 trial with teprotumumab, the drug improved both inflammatory signs and symptoms (as measured by CAS) and proptosis as compared with placebo over the 24-week study.

Smoking has been linked to an increased risk of TED, the development of more severe forms of TED (particularly those associated with proptosis and diplopia), and to delayed responses to immunosuppressive therapies [19–21]. A study conducted in patients with mild TED found that steroid therapy did not halt the progression of TED in smokers [20]. In this current analysis, the improvement in proptosis was consistent regardless of smoking status although this post-hoc analysis is limited by the small number of smokers in the teprotumumab group.

The level of proptosis reduction produced by teprotumumab is comparable with the −3.8 mm reported in a recently published retrospective trial of 169 patients with TED (319 eyes) undergoing orbital decompression [22]. Similarly, in another recently published retrospective study of 263 patients (420 orbits) who had decompression for TED at one institution, the mean reduction in proptosis was also −3.8 mm [23]. The proptosis reduction achieved with teprotumumab (mean −3.14 [range −1.0 to −6.0] mm) at week 24 compares favorably with the proptosis reduction achieved with surgery. If the extent of the proptosis response seen with the phase 2 study is confirmed in the ongoing Phase 3 study, teprotumumab may be an alternative to orbital decompression surgery without its associated complications, such as strabismus, which can occur in up to 33% of patients postoperatively [22].

## Conclusion

Teprotumumab reduced proptosis significantly beginning at 6 weeks of treatment and over the course of 24 weeks as compared with placebo, and all patients had some degree of improvement. The rapid reduction of CAS and proptosis positions teprotumumab as a promising candidate for the treatment of active TED. A Phase 3 trial with teprotumumab is currently ongoing to confirm the efficacy of teprotumumab in active TED, using proptosis as the primary outcome (NCT03298867).
